# Modification of Bacteriophages to Increase Their Association with Lung Epithelium Cells In Vitro

**DOI:** 10.3390/ph14040308

**Published:** 2021-04-01

**Authors:** Aurelija M. Grigonyte, Alexia Hapeshi, Chrystala Constantinidou, Andrew Millard

**Affiliations:** 1 Warwick Integrative Synthetic Biology Centre (WISB) and School of Life Sciences, University of Warwick, Coventry CV4 7AL, UK; grigonyt@ualberta.ca; 2 Department of Chemistry, University of Warwick, Coventry CV4 7AL, UK; A.Hapeshi@warwick.ac.uk; 3 Warwick Medical School, University of Warwick, Gibbet Hill Road, Coventry CV4 7AL, UK; C.I.Constantinidou@warwick.ac.uk; 4 Department of Genetics and Genome Biology, University of Leicester, University Road, Leicester LE1 7RH, UK

**Keywords:** phage therapy, bacteriophage T7, marker-based engineering, homing peptide, synthetic biology

## Abstract

There is currently a renaissance in research on bacteriophages as alternatives to antibiotics. Phage specificity to their bacterial host, in addition to a plethora of other advantages, makes them ideal candidates for a broad range of applications, including bacterial detection, drug delivery, and phage therapy in particular. One issue obstructing phage efficiency in phage therapy settings is their poor localization to the site of infection in the human body. Here, we engineered phage T7 with lung tissue targeting homing peptides. We then used in vitro studies to demonstrate that the engineered T7 phages had a more significant association with the lung epithelium cells than wild-type T7. In addition, we showed that, in general, there was a trend of increased association of engineered phages with the lung epithelium cells but not mouse fibroblast cells, allowing for targeted tissue specificity. These results indicate that appending phages with homing peptides would potentially allow for greater phage concentrations and greater efficacy at the infection site.

## 1. Introduction

The global antimicrobial resistance (AMR) crisis presents a serious threat to human and animal health [1,2,3]. The overuse of antibiotics and the deceleration of antibiotic discovery lead to the evolution of multidrug-resistant and pan-drug-resistant bacteria [4,5,6,7,8]. As such, novel therapeutic tools are required to avoid a post-antibiotic era in which minor injuries and common infections would be deadly [9]. The United States National Institutes of Health and reports commissioned by the Dept of Health (UK) and Wellcome Trust have identified bacteriophages as a promising alternative for combatting microbial resistance [10,11].

Bacteriophages (phages) are viruses that infect and can kill specific bacteria. Due to their versatile nature, phages have been used for a variety of applications, including bacterial control, vectors for gene therapy, recombinant protein production, pathogen detection, and therapy [12,13,14,15,16,17,18,19]. The use of phages as antimicrobial agents has several advantages over traditional antibiotics. Phages have a narrow spectrum of activity, which prevents the disturbance and dysregulation of the entire microbiome often associated with the broad activity of antibiotics against the commensal community in addition to pathogenic bacteria [20,21,22,23,24,25]. Phages do not cause adverse reactions generally related to the use of antibiotics such as nephrotoxicity, neurotoxicity, cardiotoxicity, hepatotoxicity, and a number of hematological and gastrointestinal complications [26,27,28,29]. In addition, new phages are relatively quickly discovered due to their vast abundance and diversity, capable of self-replication based on host availability (auto ‘dosing’), and contain individual components (e.g., lysins) that can have antimicrobial effects [30,31,32,33,34,35]. However, an effective phage therapy treatment of infection requires sufficient exposure to the phage at the site of bacterial invasion (a tissue or an organ) [35,36]. The dissemination of phages throughout the body to different organs can vary dramatically. Studies in mice have shown that, after three hours of intraperitoneal injection of phage suspension containing 2 × 10^10^ pfu/mL, phage concentrations in the kidney, spleen, lung, and liver were reduced to 1 × 10^4^ pfu/mL and lower concentrations within the next twelve hours [37,38]. A threshold of at least 10^7^ phages/mL has been predicted to be required for sufficient clearance of bacterial infection [39]. Therefore, a very high starting dose may be needed for effective treatment. In addition, the persistence of phage within the blood is also another variable that needs to be considered. The persistence of phage T3 is known to be greater than that of phages lambda, P22, and T7, for instance [40]. Increased persistence makes phage more attractive for therapy. The modification of phage capsids by the addition of peptides can result in reduced persistence as has been found for phage T4 [41]. If this generally applies to all phages is not known.

Reaching a high enough phage titer at the tissue or organ of interest is likely to be a limiting factor in the successful widespread adoption of phage therapy [35,36]. As such, targeted organ or tissue delivery of phages may be required to achieve greater phage concentration at a site of infection. The implementation of targeted phage delivery requires a better understanding of the phages’ role in the human body. Phage particles are the most numerous group in the human virome [42,43,44]. Due to the cell surface and intracellular machinery differences between eukaryotic and bacterial hosts, phages cannot infect and replicate in eukaryotic cells. However, phage particles can penetrate and move around the human body as well as the bodies of higher vertebrates [45,46,47]. The exact mechanism of phage distribution in the human body has not been fully elucidated. As such, a variety of routes have been postulated over the years. Given that the gut contains the largest number of phages in humans, the gut is the most likely primary phage source [48,49]. One of the routes proposed is that of “leaky gut,” where phages could be released into the bloodstream due to punctured vasculature [50,51]. Another proposed mechanism suggests phage entry into a mammalian cell via infected bacterial cell that is later engulfed by epithelial cells, referred to as the “Trojan horse” mechanism. A generalized mechanism for phage translocation into and across confluent epithelial cell layers was recently suggested [52]. The study showed that phage transcytosis across confluent cell layers had significant preferential directionality for apical-to-basal transport [52].

With the potential for phages to move throughout the body and predicted minimum concentrations of phages required at a site of infection, strategies are needed to make phages tissue-specific. The human body’s heterogeneity could be exploited to inform novel approaches for targeted tissue specificity. Previous studies have shown that vascular endothelium and epithelium cells express tissue specific-markers [53,54,55,56,57,58,59,60,61]. Specifically, the vascular endothelium and epithelium of healthy or diseased organs possess organ-specific molecular markers or receptors, and ligands binding to them are referred to as “homing peptides (HP)” [57,62]. In vivo phage display technology has been used for tissue-specific marker recognition to identify extensive molecular differences in the vasculature [63]. Multiple peptides targeting normal and tumor blood vessels or tumor lymphatic vessels have been isolated [64,65,66,67,68,69]. In addition, homing peptides targeting the vasculature of various organs, including the lung, heart, prostate, skeletal and cardiac muscle, and adipose tissue, have been identified [64,65,70,71,72]. Homing peptides have been exploited for targeting drug molecules, liposomes, and inorganic nanoparticles to tissues [62,73]. Their use has increased the targeting specificity and efficacy of drug delivery and reduced drug-associated side effects, presumably by lowering unspecific targeting of healthy tissues and organs [74,75]. Arming phages with homing peptides, a technique previously applied to other nanoparticles, would potentially allow greater phage concentrations and greater efficacy at the infection site [73].

Here, we aimed to engineer phages with peptides that have previously been shown to be specific to lung epithelial and endothelial cells. We then tested this in vitro for a greater association of engineered phages than the wild-type with two different cell lines, demonstrating engineering of phages with peptides can provide increased cell-specific association.

## 2. Results

### 2.1. Association of T4-Like and T7 with Lung Epithelial Cells

**Figure 1:**
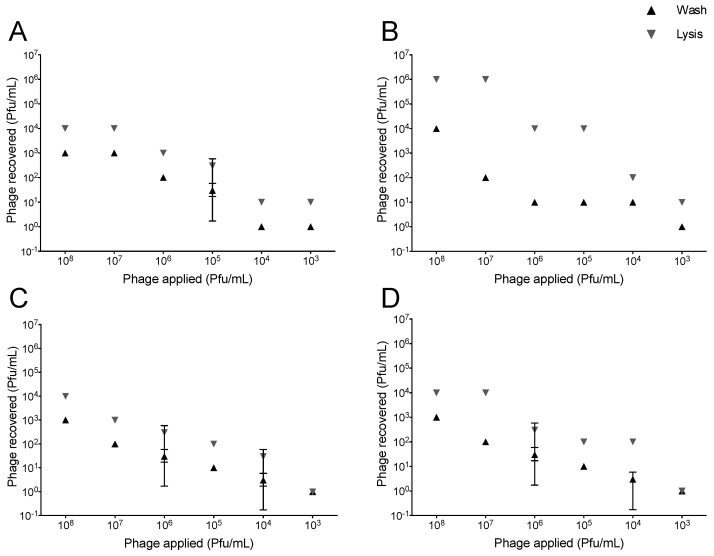
Recovery of phages SLUR96 and T7 from association assay on A549 lung epithelial cells. Phages SLUR96 and T7 were incubated with A549 cells (80–90% confluence) at 10^3^ to 10^8^ pfu/mL for one or four hours. A549 cells were treated (washed and lysed), followed by an enumeration of phages for both wash and lysis treatments using spot assay. The pfu/mL for each treatment was determined against *E. coli* BW25113. Phage species and incubation times: (**A**) Slur96, one hour, (**B**) Slur96, four hours, (**C**) T7, one hour, and (**D**) T7 four hours. (Replicates *n* = 2 for all conditions).

To determine the association of wild-type phages with cells, we developed an assay to assess phages’ association with A549 lung epithelial cells. We tested this system using phages vB_Eco_SLUR96 (SLUR96) and T7. Phage SLUR96 is a T4-like phage recently isolated on *E. coli* MG1655. Phages were applied at six different concentrations ranging from 10^8^ to 10^3^ pfu/mL and quantified after one and four hours of incubation by plating with their bacterial host. The association assay (Methods 4.4) allows for the identification of loosely bound phages (wash step) and the determination of phages that are either attached firmly to the cell surface or that were internalized by the mammalian cell (lysis step). The number of phages recovered after both wash and lysis steps were dose-dependent (Figure 1). As the concentration of applied phage was sequentially reduced (10^8^ to 10^3^ pfu/mL), the recovery decreased accordingly. The concentration of 10^3^ pfu/mL represents the limit of detection for the assay. The highest concentration of total phages recovered after one hour of incubation after both wash and lysis was approximately 10^4^ pfu/mL for phages SLUR96 and T7 (Figure 1A,C). Increasing the incubation period from one to four hours resulted in ~100 times greater phage SLUR96 recovery than T7, indicating greater SLUR96 association with A549 lung epithelial cells.

### 2.2. Modification of Phage Capsids with “Homing” Peptides

Having established that we could assay the association of bacteriophages with epithelial cells, we then sought to modify a phage to increase this association. Although mutants have been made for both T7 and T4-like phages, we chose to focus on phage T7 due to more well developed genetic engineering system [76,77,78,79,80]. Three peptides that have previously been identified as “homing” peptides for lung vasculature were selected [70,81]. GFE-1 and GFE-2 were shown to bind to membrane dipeptidase receptor found on both epithelial and endothelial lung tissue cells [70,82,83]. The third “homing” peptide was a receptor-binding domain of metadherin (MTDH), a cell surface protein in breast tumors shown to home to lung endothelial cells [81]. The receptor for the MTDH domain (will be referred to as MTDH) homing peptide is unknown [81]. All three peptides are short, constituting 8 to 63 amino acids in length (Table S1). Using homologous recombination, the genes encoding the major (10A) and minor (10B) capsid proteins were modified to incorporate the peptides in the C-terminus of the 10A and 10B proteins (Methods 4.2 and 4.3). The capsid of phage T7 is usually made of both major (10A) and minor (10B) proteins at a ratio of ~10:1. Thus, the insertion of the different subunits should provide different levels of protein expression. The resulting six engineered T7 phages had three homing peptides inserted in the 10A or 10B capsid protein (Table 1).

### 2.3. Increased Association of Phages with A549 Human Lung Epithelial Cells

**Figure 2:**
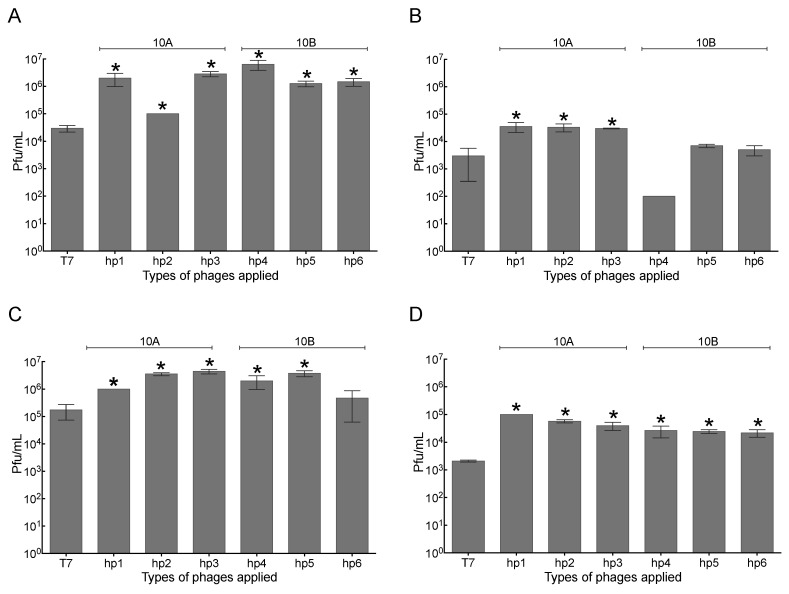
Quantification of wild-type phage T7 and engineered T7 recovery on A549 human lung epithelial cells. Each phage was incubated with A549 cells (80–90% confluence) at 2 × 10^8^ pfu/mL for either one or four hours. A549 cells were then washed and lysed followed by phage enumeration in each case: (**A**) wash step after one hour of incubation, (**B**) lysis step after one hour of incubation, (**C**) wash step after four hours of incubation, (**D**) lysis step after four hours of incubation. The pfu/mL for each sample was determined on the *E. coli* BW25113 strain. * *p* < 0.05, when compared to wild-type T7. The error bars come from replicates (*n* = 3) and, in some instances, too small to be represented.

The association of wild-type T7 and modified T7- phage particles was measured using the previously established assay with A549 lung epithelial cells. After the wash step and one hour of incubation, all modified phages (one-way ANOVA, Post Hoc, *p* < 0.05) showed significantly greater numbers of phages recovered to that of wild-type phage T7 (Figure 2A). The number of phage particles recovered after the lysis step was significantly higher for all T7-hp1–T7-hp3 mutants than wild-type T7 (Figure 2B). After the wash step and four hours of incubation, there was an increased recovery of all modified phages except from T7-hp6 (one-way ANOVA, Post Hoc, *p* < 0.05) than wild-type phage T7 (Figure 2C). After the lysis step and four hours of incubation, the recovery of all T7-hp phages was significant for all six mutants compared to wild-type phage T7 (one-way ANOVA, Post Hoc, *p* < 0.05) (Figure 2D). The four-hour incubation period showed that all three peptides, regardless of the insertion in the major or minor subunits, resulted in a stronger association with A549 cells than wild-type phage T7. Furthermore, the insertion of peptides in the major capsid subunit (10A) resulted in increased recovery of phages in all conditions tested. After one hour of incubation and the wash step, T7-hp4 showed an approximately 213-fold increase compared to wild-type T7, whereas after the lysis step, T7-hp1 was the best performing mutant with approximately 13-fold greater phage recovery numbers when compared with wild-type T7.

### 2.4. Association of Phage with 3T3 Mouse Embryonic Fibroblast Cells

Having established that modified phage T7 formed a stronger association with epithelial lung cells, we next tested these phages against mouse 3T3 embryonic fibroblast cells, which would not be expected to have increased association based on the presence of the incorporated peptides. The association of wild-type and modified T7-hp phages was assayed against embryonic fibroblast 3T3 cells. There was a greater association of wild-type T7 with 3T3 cells than A549 cells (*p* < 0.05). One-way ANOVA was carried out to identify the differences between wild-type T7 and T7-hps on 3T3 cells.

**Figure 3:**
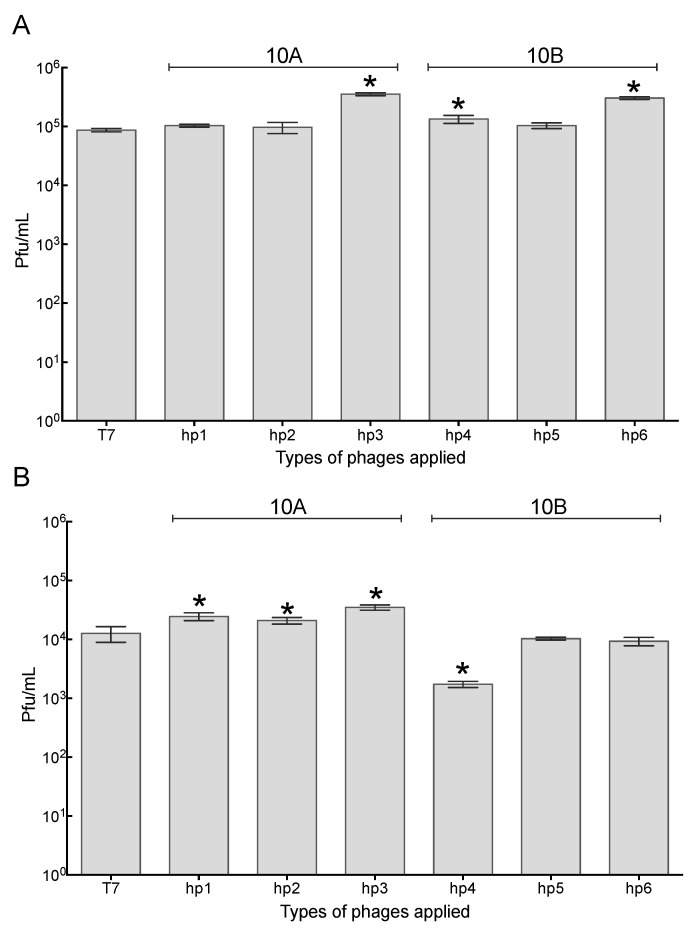
Quantification of wild-type phage T7 and engineered T7 recovery on 3T3 mouse embryonic fibroblast cells. Each phage was incubated with 3T3 cells (80–90% confluence) at 2 × 10^8^ pfu/mL for one hour. 3T3 cells were then washed and lysed, followed by phage enumeration in each case: (**A**) wash step after one hour of incubation, (**B**) lysis step after one hour of incubation. The pfu/mL for each sample was determined on *E. coli* BW25113. * *p* < 0.05, when compared to T7. The error bars come from replicates (*n* = 3).

The incubation with 3T3 embryonic fibroblast cells resulted in increased recovery of T7-hp3, T7-hp4, and T7-6 when compared to wild-type T7 after the wash step (*p* < 0.05) (Figure 3A). Although there was significantly increased recovery of the engineered phages, the greatest recovery was only a 4-fold increase for T7-hp3; in comparison, in A549 cells, the same peptide resulted in a ~97-fold increase. After the lysis step, phages T7-hp1–T7-hp3 resulted in significantly higher recovery numbers than wild-type T7 (*p* < 0.05). Although the recovery increased, the largest increase was a 3-fold increase for T7-hp3, compared to wild-type T7. Intriguingly, T7-hp4 showed a lower recovery number than wild-type T7 (Figure 3B) (*p* < 0.05).

## 3. Discussion

A limitation for phage therapy has been identified as achieving a high enough phage titer at an infection site [36,39,84]. One way to overcome this is to modify phages to form a stronger association with particular eukaryotic cell types, which we aimed to do. We first developed an assay that determines the association of phages with eukaryotic cells. As a proof of principle study, we chose lung epithelium cells for our phage association assay, based on existing literature on the potential for homing peptides to target this cell type [70,85,86]. With respect to treating lung infections, phages could be potentially administered via an intravenous route or through inhalation of aerosols. However, lung cells offered a basis to develop a system.

Using phages SLUR96 and T7, we demonstrated a time and dose-dependent response. The assay allowed the differentiation between weakly and more strongly associated interactions of phages with eukaryotic cells. Phage T7 was then engineered to incorporate the sequence of “homing” peptides GFE-1, GFE-2, and MTDH into the genes encoding the capsid protein’s major (10A) and minor subunits (10B). Viable mutants were obtained for all six possible combinations. Previous use of phage T7 as a phage display system has demonstrated that the capsid can tolerate several modifications, in both minor and major subunits, with the insertion of very large peptides preferable in the minor subunit [87,88]. Given the small nature of the peptide used in this study, it is not surprising that it was possible to incorporate them in both the major and minor capsid subunits. Of the six mutants constructed, phages T7-hp1–T7-hp3 showed the greatest promise with significantly greater numbers of phages recovered across all conditions tested, with a 3–97-fold increase. Phages T7-hp1–T7 had three homing peptides inserted into the major capsid protein (10A). Given the standard ratio of ~9:1 of major to minor capsid proteins in the T7 virion, this suggests that increasing the number of displayed peptides increases the association with A549 cells. T7-hp3 showed the greatest number of phages recovered from the three peptides after the wash step, with ~97 fold increase compared to wild-type T7. The difference in the association could arise from the expression levels of each homing peptide receptor on the lung vasculature. Both GFE1/2 peptides bind to the membrane dipeptidase receptor, whereas the target receptor of the MTDH on lung vasculature has not been identified [70,81]. Contrary to the other two peptides (GFE-1/2), the MTDH domain was overexpressed in breast tumors and bound to lung vasculature [63,74,75]. Therefore, it is important to note that the result of T7-hp3 showing the greatest association with lung tissue after the wash step could be due to the use of the A549 cell line, which is derived from carcinoma and potentially contains more MTDH binding receptors compared to healthy (non-carcinoma) cells. Further in vivo experimental work is required to determine if the result may differ for healthy lung epithelium cells.

Based on the encouraging results on A549 cells, the same modified phages were tested against 3T3 embryonic fibroblast cells, for which no increase in the association would be expected by the incorporation of peptides thought to be specific to A549 cells. Wild-type T7 showed a significantly greater association with the difference between the 3T3 cells when compared to A549 cells. The preferential binding of wild-type T7 to different mammalian cells could be a potential drawback when trying to increase the association using a specific tissue targeting peptide. A larger-scale comparison between multiple mammalian tissues is required to determine if a preferential binding is valid across different systems of the human body.

When comparing the association of modified phages to wild-type T7 on 3T3 cells, the general pattern showed some increased association of modified phages compared to wild-type T7 after both wash and lysis steps. Increased association on 3T3 cells was unexpected and might suggest that modifications do not increase cell-specific association. However, the fold change in recovery was markedly higher for all peptides on A549 cells than 3T3 cells. The highest increase in recovery was ~213-fold on A549 cells, compared to ~6-fold on 3T3 cells, suggesting the peptides cause an increase in association with a specific cell type.

What form of association occurs was not determined in this study. The use of a wash step and lysis step allowed the differentiation of weak and strong association of phages with cells. However, the nature of the stronger association is not clear. The assay we used will not discriminate between phages bound firmly to the cell surface or phages that have been internalized by the cell. A recent in vitro study demonstrated rapid and directional transcytosis of different phages across a monolayer of the lung, gut, kidney, liver, and brain cells [52]. The transcytosis process was demonstrated by applying ~10^8^ pfu/mL phage T4 to a monolayer of A549 lung epithelial cells in a contralateral chamber and two incubation hours before the recovery of ~10^4^ pfu/mL phage from the basal fraction [52]. Consequently, it was shown that phage transcytosis across cell layers occurred after two hours of incubation. In comparison to our study, when ~10^8^ pfu/mL of phage SLUR96 (T4-like) was applied to a monolayer of A549, a similar recovery of ~10^4^ pfu/mL of phage after the lysis step and one hour of incubation occurred. Thus, the recovery of phages collected after the lysis step likely represents phages that have been internalized by the lung tissue epithelium cells.

Transcytosis of bacteriophages has been suggested as a general mechanism for phage movement across confluent epithelial cells accounting for phage abundance in the human body. Phage transcytosis across cell layers has a preferential directionality for apical-to-basolateral transport. However, phage interactions with different eukaryotic cells have not been elucidated to date. Present evidence highlights non-specific phage and eukaryotic cell interactions. In one instance, lytic phages were found to aid the intracellular killing of engulfed methicillin-resistant *Staphylococcus aureus* (MRSA) by murine macrophages [89]. The phage was carried into macrophages while bound to bacteria and showed reduced cytotoxic damage caused by MRSA, enhancing the bactericidal killing potential of phagocytic cells [89]. In another study, lytic phage vB_SauM_JS25 penetrated bovine mammary epithelial cells allowing for effective killing of intracellular *Staphylococcus aureus* [90]. Further examples exist, where phages bind preferentially to a given receptor on a mammalian cell [91,92]. *Escherichia coli* phage PK1A2 has been shown to interact with various cell types expressing polysialic acid [92]. In another instance, T4 and HAP1 phages were proposed to bind to integrin *β*3 via the Lys-Gly-Asp motif of phage protein 24 in both HS294T and A549 cells [92]. Given the limited knowledge of how exactly specific phage-tissue interactions occur, the use of “homing” peptides could make these interactions more specific. Homing peptides have previously been used to enhance treatment specificity and drug concentrations at a target site [93,94,95,96]. In all instances, a drug tagged with a homing peptide resulted in higher amounts at the target site when compared with the non-tagged drug [93,94,95,96]. Most of these previous examples have been used for cancer rather than bacterial infections. The engineering of phages with cell-specific peptides suggests that in principle the approach could be used to treat bacterial infections. It was possible to demonstrate in vitro that the display of the “homing” peptide MTDH in the major capsid protein increased the association of phages with A549 cells. Although the same peptide resulted in a significant increase with 3T3 cells, the scale of the increased association was far higher in A549 cells than 3T3 cells. If this association is internal or external to the cell is not clear at this stage and will require further investigation. In addition, whether this increased association is replicated in vivo still needs to be confirmed.

Currently, how long phages are retained in the lungs is not clear. The limited previous research in a mouse model suggests phages are cleared within 72 h [97]. Once an infection is cleared by phages, it might be expected for phages also to be cleared. The effect of introducing peptides into the phage T7 capsid on the phage retention time in the body is also not known. It remains to be determined if engineering phages with homing peptides causes a decrease in retention time, like the modification of the T4 capsid [41]. Our results solely focus on the interaction of phages with different cell types. Whilst the phages remain infective and viable in culture, the effects of phage modification remain to be addressed.

However, the data generated in this study suggest that the engineering of phages to improve their tissue-specificity is a promising avenue of research that should be pursued further. In addition to the therapeutic applications of phages engineered with peptides to target bacterial infections, there is also the potential to treat tumors. Previously, genetically modified phages have been used to target glioblastoma, using phages as a delivery vehicle [98]. Given A549 cells are derived from cancer cells, there is the potential to further exploit engineered phage T7 as a drug delivery vehicle in a similar manner.

## 4. Materials and Methods

### 4.1. Bacterial Strains, Phage Stocks, Tissue Culture Cell Lines, and Growth Conditions

*E. coli* BW25113, *E. coli* BW25113 Δ*trxA* and *E. coli* MG1655 were grown in LB (10 g tryptone, 5 g yeast extract, 10 g NaCl, in 1 L distilled water [dH_2_O], (Oxoid, Basingstoke, UK)) at 37 °C with shaking overnight (200 rpm) and used to propagate, quantify, for phages Slur96 (T4 like phage, genome 95% similar to the wild-type T4 isolated in Millard lab, University of Leicester, ENA Project submission number: PRJEB43467 and T7 (Richardson Lab, Harvard University), and carry out homologous recombination and marker-based selection for phage T7. Phages SLUR96 and T7 were propagated by inoculating *E. coli* culture at an OD 600 nm of 0.4 and 0.3, respectively. Phage lysates were filtered using a 0.22 μm pore size filter (Sartorius, Dublin, Ireland) and stored at 4 °C. Phage enumeration was carried out using a standard double agar overlay plaque assay or spot assays, as previously described [99]. A549 adenocarcinomic human alveolar basal epithelial and 3T3 mouse embryonic fibroblast 3T3 cell lines were grown at 37 °C and 5% CO_2_. A549 and 3T3 cell lines were grown in DMEM (potassium chloride (KCl)—400 mg/L, sodium bicarbonate (NaHCO_3_)—3700 mg/L, sodium chloride (NaCl)—6400 mg/L, Thermo Fisher Scientific, Waltham, MA, USA) medium with 10% fetal bovine serum (FBS, Thermo Fisher Scientific).

### 4.2. Design of Homologous Recombination Vectors for Homing Peptide Insertion

The six vectors used in phage T7 homologous recombination (HR) were synthesized by (IDT) to have HR arms of 97–99 bp flanking the homing peptide, an RBS site, and the *trxA* gene in a pSMART backbone (IDT) (Table S2). The vectors were designed to allow for homing peptide insertion after 10A and 10B capsid proteins after 345 and 398 aa, respectively. The vectors constructed were transformed into *E. coli* BW25113 Δ*trxA* cells followed by homologous recombination and marker-based selection of T7-hp phages [76].

### 4.3. Selection of T7 Mutants

The selection of T7 mutants was carried out as previously described in detail [76]. Briefly, *E. coli* BW25113 Δ*trxA* strain containing an HR plasmid (Method 4.2 and Table S2) was grown until it reached an 0.3 at OD600 nm, whereby phage T7 was then added at MOI ~0.01. The culture/phage mixture was then incubated at 37 °C while shaking (200 rpm) for 3 h. The lysate was then filtered through a 0.22 μm pore size filter and stored at 4 °C until further use. For marker-based selection, the lysate was plated on *E. coli* BW25113 Δ*trxA* strain containing (CITE marker-based). The resulting plaques were isolated and screened on *E. coli* BW25113 Δ*trxA* for additional two rounds, followed by propagation on *E. coli* BW25113 Δ*trxA* strain. Following homologous recombination and selection, plaques were picked from plates and resuspended in 1 mL of SM buffer. PCR followed by bidirectional Sanger sequencing was used to confirm the mutants (Table S3).

### 4.4. Phage Association Assay

Tissue culture 6-well plates (Falcon) were used for all adsorption and absorption experiments. All cells were seeded at a density of 1 × 10^6^ per well and allowed to grow to confluence (3 to 5 days). Phages Slur96 and T7 were applied with DMEM medium and incubated with cells for either one or four hours. The medium (pre-wash) was removed, followed by the addition of PBS while gently pipetting up and down. The PBS addition was repeated twice, and the resulting medium (wash step) was collected. Then, 0.5% saponin (Sigma Aldrich, St. Louis, MO, USA) was added to each well, and the plate was incubated for 15 min at 37 °C. After the incubation, the saponin in each well was pipetted up and down, followed by sample collection (lysis step). The samples collected during the pre-wash, wash, and lysis steps were used for phage quantification by plating with their bacterial host.

## Figures and Tables

**Table 1:** Summary of T7-hp mutants generated in this study indicates the capsid protein and homing peptide used to engineer each mutant.

T7 Mutant	Structural Protein Subunit	Peptide
T7-hp1	A	GFE-1
T7-hp2	A	GFE-2
T7-hp3	A	MTDH
T7-hp4	B	GFE-1
T7-hp5	B	GFE-2
T7-hp6	B	MTDH

## Data Availability

The genome data presented in this is available under project number PRJEB43467.

## Supplementary Materials

The following are available online at https://www.mdpi.com/article/10.3390/ph14040308/s1, Table S1: Sequences for the homing peptides used in this study; Table S2: Sequences used for homologous recombination vector synthesis; Table S3: Primers used in this study.

## References

[1] The United States Centers for Disease Control and Prevention (2008). GET SMART: Know When Antibiotics Work. AJN.

[2] Shallcross L.J., Davies S.C. (2014). The World Health Assembly resolution on antimicrobial resistance. J. Antimicrob. Chemother..

[3] World Health Organization (2006). Antimicrobial Use in Aquaculture and Antimicrobial Resistance. Aeromonas Resist. How It Eff. Hum..

[4] Levy S.B., Bergman M.M. (2003). The Antibiotic Paradox: How the Misuse of Antibiotics Destroys Their Curative Powers, 2nd Edition. Clin. Infect. Dis..

[5] Nathan C. (2004). Antibiotics at the crossroads. Nature.

[6] Yewale V.N. (2014). Antimicrobial resistance—A ticking bomb!. Indian Pediatr..

[7] Schlesinger L.A., Kotter J.P. (2008). Choosing Strategies for Change—HBR.org. Harv. Bus. Rev..

[8] Moellering R.C. (2010). NDM-1—A Cause for Worldwide Concern. N. Engl. J. Med..

[9] Alanis A.J. (2005). Resistance to antibiotics: Are we in the post-antibiotic era?. Arch. Med. Res..

[10] Czaplewski L., Bax R., Clokie M., Dawson M., Fairhead H., Fischetti V.A., Foster S., Gilmore B.F., Hancock R.E.W., Harper D. (2016). Alternatives to antibiotics-a pipeline portfolio review. Lancet Infect. Dis..

[11] (2014). NIAID’s Antibacterial Resistance Program: Current Status and Future Directions. NIAID.

[12] Abedon S.T., García P., Mullany P., Aminov R. (2017). Editorial: Phage therapy: Past, present and future. Front. Microbiol..

[13] Roach D.R., Leung C.Y., Henry M., Morello E., Singh D., Di Santo J.P., Weitz J.S., Debarbieux L. (2017). Synergy between the Host Immune System and Bacteriophage Is Essential for Successful Phage Therapy against an Acute Respiratory Pathogen. Cell Host Microbe.

[14] Gelman D., Eisenkraft A., Chanishvili N., Nachman D., Coppenhagem Glazer S., Hazan R. (2018). The history and promising future of phage therapy in the military service. J. Trauma Acute Care Surg..

[15] Van der Merwe R.G., Warren R.M., Sampson S.L., Gey van Pittius N.C. (2014). Phage-based detection of bacterial pathogens. Analyst.

[16] Moye Z.D., Woolston J., Sulakvelidze A. (2018). Bacteriophage applications for food production and processing. Viruses.

[17] Coffey B., Mills S., Coffey A., McAuliffe O., Paul Ross R. (2010). Phage and their lysins as biocontrol agents for food safety applications. Annu. Rev. Food Sci. Technol..

[18] Lone A., Anany H., Hakeem M., Aguis L., Avdjian A.C., Bouget M., Atashi A., Brovko L., Rochefort D., Griffiths M.W. (2016). Development of prototypes of bioactive packaging materials based on immobilized bacteriophages for control of growth of bacterial pathogens in foods. Int. J. Food Microbiol..

[19] Wang C., Sauvageau D., Elias A. (2016). Immobilization of Active Bacteriophages on Polyhydroxyalkanoate Surfaces. ACS Appl. Mater. Interfaces.

[20] Zhang S., Chen D.C., Chen L.M. (2019). Facing a new challenge: The adverse effects of antibiotics on gut microbiota and host immunity. Chin. Med. J..

[21] Willing B.P., Russell S.L., Finlay B.B. (2011). Shifting the balance: Antibiotic effects on host-microbiota mutualism. Nat. Rev. Microbiol..

[22] Becattini S., Taur Y., Pamer E.G. (2016). Antibiotic-Induced Changes in the Intestinal Microbiota and Disease. Trends Mol. Med..

[23] Robak O.H., Heimesaat M.M., Kruglov A.A., Prepens S., Ninnemann J., Gutbier B., Reppe K., Hochrein H., Suter M., Kirschning C.J. (2018). Antibiotic treatment-induced secondary IgA deficiency enhances susceptibility to Pseudomonas aeruginosa pneumonia. J. Clin. Investig..

[24] Chibani-Chennoufi S., Sidoti J., Bruttin A., Kutter E., Sarker S., Brüssow H. (2004). In vitro and in vivo bacteriolytic activities of Escherichia coli phages: Implications for phage therapy. Antimicrob. Agents Chemother..

[25] Sarker S.A., McCallin S., Barretto C., Berger B., Pittet A.C., Sultana S., Krause L., Huq S., Bibiloni R., Bruttin A. (2012). Oral T4-like phage cocktail application to healthy adult volunteers from Bangladesh. Virology.

[26] Granowitz E.V., Brown R.B. (2008). Antibiotic Adverse Reactions and Drug Interactions. Crit. Care Clin..

[27] Morales-Alvarez M.C. (2020). Nephrotoxicity of Antimicrobials and Antibiotics. Adv. Chronic Kidney Dis..

[28] Grill M.F., Maganti R.K. (2011). Neurotoxic effects associated with antibiotic use: Management considerations. Br. J. Clin. Pharmacol..

[29] Lu Z.K., Yuan J., Li M., Sutton S.S., Rao G.A., Jacob S., Bennett C.L. (2015). Cardiac risks associated with antibiotics: Azithromycin and levofloxacin. Expert Opin. Drug Saf..

[30] Clokie M.R.J., Kropinski A.M. (2009). Bacteriophages: Methods and protocols Volume 1: Isolation, Characterization, and Interactions.

[31] Carlton R.M. (1999). Phage therapy: Past history and future prospects. Arch. Immunol. Ther. Exp..

[32] Abedon S.T. (2015). Bacteriophage secondary infection. Virol. Sin..

[33] Capparelli R., Nocerino N., Iannaccone M., Ercolini D., Parlato M., Chiara M., Iannelli D. (2010). Bacteriophage therapy of Salmonella enterica: A fresh appraisal of bacteriophage therapy. J. Infect. Dis..

[34] Skurnik M., Strauch E. (2006). Phage therapy: Facts and fiction. Int. J. Med. Microbiol..

[35] Abedon S.T., Thomas-Abedon C. (2010). Phage therapy pharmacology. Curr. Pharm. Biotechnol..

[36] Górski A., Dąbrowska K., Hodyra-Stefaniak K., Borysowski J., Międzybrodzki R., Weber-Dąbrowska B. (2015). Phages targeting infected tissues: Novel approach to phage therapy. Future Microbiol..

[37] Keller R., Engley F.B. (1958). Fate of Bacteriophage Particles Introduced into Mice by Various Routes. Proc. Soc. Exp. Biol. Med..

[38] Bogovazova G.G., Voroshilova N.N., Bondarenko V.M. (1991). The efficacy of Klebsiella pneumoniae bacteriophage in the therapy of experimental Klebsiella infection. Zh. Mikrobiol. Epidemiol. Immunobiol..

[39] Abedon S.T., Międzybrodzki J.B.R., Górski A. (2014). Bacteriophages as Drugs: The Pharmacology of Phage Therapy. Phage Therapy: Current Research and Applications.

[40] Serwer P., Wright E.T., Lee J.C. (2019). High murine blood persistence of phage T3 and suggested strategy for phage therapy. BMC Res. Notes.

[41] Hodyra-Stefaniak K., Lahutta K., Majewska J., Kaźmierczak Z., Lecion D., Harhala M., Kęska W., Owczarek B., Jończyk-Matysiak E., Kłopot A. (2019). Bacteriophages engineered to display foreign peptides may become short-circulating phages. Microb. Biotechnol..

[42] Gregory A.C., Zablocki O., Howell A., Bolduc B., Sullivan M.B. (2019). The human gut virome database. bioRxiv.

[43] Hoyles L., McCartney A.L., Neve H., Gibson G.R., Sanderson J.D., Heller K.J., van Sinderen D. (2014). Characterization of virus-like particles associated with the human faecal and caecal microbiota. Res. Microbiol..

[44] Shkoporov A.N., Ryan F.J., Draper L.A., Forde A., Stockdale S.R., Daly K.M., McDonnell S.A., Nolan J.A., Sutton T.D.S., Dalmasso M. (2018). Reproducible protocols for metagenomic analysis of human faecal phageomes. Microbiome.

[45] Dabrowska K., Switała-Jelen K., Opolski A., Weber-Dabrowska B., Gorski A. (2005). A review: Bacteriophage penetration in vertebrates. J. Appl. Microbiol..

[46] Górski A., Wazna E., Dąbrowska B.W., Dąbrowska K., Świtała-Jeleń K., Miȩdzybrodzki R. (2006). Bacteriophage translocation. FEMS Immunol. Med. Microbiol..

[47] Weber-Dabrowska B., Dabrowski M., Slopek S. (1987). Studies on bacteriophage penetration in patients subjected to phage therapy. Arch. Immunol. Ther. Exp..

[48] Sutton T.D.S., Hill C. (2019). Gut Bacteriophage: Current Understanding and Challenges. Front. Endocrinol..

[49] Navarro F., Muniesa M. (2017). Phages in the human body. Front. Microbiol..

[50] König J., Wells J., Cani P.D., García-Ródenas C.L., MacDonald T., Mercenier A., Whyte J., Troost F., Brummer R.J. (2016). Human intestinal barrier function in health and disease. Clin. Transl. Gastroenterol..

[51] Huh H., Wong S., St. Jean J., Slavcev R. (2019). Bacteriophage interactions with mammalian tissue: Therapeutic applications. Adv. Drug Deliv. Rev..

[52] Nguyen S., Baker K., Padman B.S., Patwa R., Dunstan R.A., Weston T.A., Schlosser K., Bailey B., Lithgow T., Lazarou M. (2017). Bacteriophage transcytosis provides a mechanism to cross epithelial cell layers. MBio.

[53] Aird W.C. (2012). Endothelial cell heterogeneity. Cold Spring Harb. Perspect. Med..

[54] Garlanda C., Dejana E. (1997). Heterogeneity of endothelial cells: Specific markers. Arterioscler. Thromb. Vasc. Biol..

[55] Aird W.C. (2007). Phenotypic heterogeneity of the endothelium: II. Representative vascular beds. Circ. Res..

[56] Rajotte D., Arap W., Hagedorn M., Koivunen E., Pasqualini R., Ruoslahti E. (1998). Molecular Heterogeneity of the Vascular Endothelium Revealed by In Vivo Phage Display. J. Clin. Investig.

[57] Costantini T.W., Putnam J.G., Sawada R., Baird A., Loomis W.H., Eliceiri B.P., Bansal V., Coimbra R. (2009). Targeting the gut barrier: Identification of a homing peptide sequence for delivery into the injured intestinal epithelial cell. Surgery.

[58] Curnis F., Arrigoni G., Sacchi A., Fischetti L., Corti A., Arap W., Pasqualini R. (2002). Differential binding of drugs containing the NGR motif to CD13 isoforms in tumor vessels, epithelia, and myeloid cells. Cancer Res..

[59] Morris C.J., Smith M.W., Griffiths P.C., McKeown N.B., Gumbleton M. (2011). Enhanced pulmonary absorption of a macromolecule through coupling to a sequence-specific phage display-derived peptide. J. Control. Release.

[60] Park S., Kim Y.J., Jon S. (2014). A high-affinity peptide for nicotinic acetylcholine receptor-α1 and its potential use in pulmonary drug delivery. J. Control. Release.

[61] Wu M., Pasula R., Smith P.A., Martin W.J. (2003). Mapping alveolar binding sites in vivo using phage peptide libraries. Gene Ther..

[62] Laakkonen P., Vuorinen K. (2010). Homing peptides as targeted delivery vehicles. Integr. Biol..

[63] Pasqualini R., Ruoslahti E. (1996). Organ targeting in vivo using phage display peptide libraries. Nature.

[64] Arap W., Haedicke W., Bernasconi M., Kain R., Rajotte D., Krajewski S., Ellerby H.M., Bredesen D.E., Pasqualini R., Ruoslahti E. (2002). Targeting the prostate for destruction through a vascular address. Proc. Natl. Acad. Sci. USA.

[65] Zhang L., Hoffman J.A., Ruoslahti E. (2005). Molecular profiling of heart endothelial cells. Circulation.

[66] Pasqualini R., Koivunen E., Ruoslahti E. (1997). αv Integrins as Receptors for Tumor Targeting by Circulating Ligands. Nat. Biotechnol..

[67] Porkka K., Laakkonen P., Hoffman J.A., Bernasconi M., Ruoslahti E. (2002). A fragment of the HMGN2 protein homes to the nuclei of tumor cells and tumor endothelial cells in vivo. Proc. Natl. Acad. Sci. USA.

[68] Laakkonen P., Akerman M.E., Biliran H., Yang M., Ferrer F., Karpanen T., Hoffman R.M., Ruoslahti E. (2004). Antitumor activity of a homing peptide that targets tumor lymphatics and tumor cells. Proc. Natl. Acad. Sci. USA.

[69] Zhang L., Giraudo E., Hoffman J.A., Hanahan D., Ruoslahti E. (2006). Lymphatic zip codes in premalignant lesions and tumors. Cancer Res..

[70] Rajotte D., Ruoslahti E. (1999). Membrane dipeptidase is the receptor for a lung-targeting peptide identified by in vivo phage display. J. Biol. Chem..

[71] Samoylova T.I., Smith B.F. (1999). Elucidation of muscle-binding peptides by phage display screening. Muscle Nerve.

[72] Kolonin M.G., Saha P.K., Chan L., Pasqualini R., Arap W. (2004). Reversal of obesity by targeted ablation of adipose tissue. Nat. Med..

[73] Ruoslahti E. (2012). Peptides as targeting elements and tissue penetration devices for nanoparticles. Adv. Mater..

[74] Ellerby H.M., Arap W., Ellerby L.M., Kain R., Andrusiak R., Del Rio G., Krajewski S., Lombardo C.R., Rao R., Ruoslahti E. (1999). Anti-cancer activity of targeted pro-apoptotic peptides. Nat. Med..

[75] Corti A., Curnis F., Rossoni G., Marcucci F., Gregorc V. (2013). Peptide-mediated targeting of cytokines to tumor vasculature: The NGR-hTNF example. BioDrugs.

[76] Grigonyte A.M., Harrison C., MacDonald P.R., Montero-Blay A., Tridgett M., Duncan J., Sagona A.P., Constantinidou C., Jaramillo A., Millard A. (2020). Comparison of CRISPR and marker-based methods for the engineering of phage T7. Viruses.

[77] Liu Y., Huang H., Wang H., Zhang Y. (2020). A novel approach for T7 bacteriophage genome integration of exogenous DNA. J. Biol. Eng..

[78] Jensen J.D., Parks A.R., Adhya S., Rattray A.J., Court D.L. (2020). λ Recombineering Used to Engineer the Genome of Phage T7. Antibiotics.

[79] Chan L.Y., Kosuri S., Endy D. (2005). Refactoring bacteriophage T7. Mol. Syst. Biol..

[80] Studier F.W. (1969). The genetics and physiology of bacteriophage T7. Virology.

[81] Brown D.M., Ruoslahti E. (2004). Metadherin, a cell surface protein in breast tumors that mediates lung metastasis. Cancer Cell.

[82] Habib G.M., Barriost R., Shi Z.Z., Lieberman M.W. (1996). Four distinct membrane-bound dipeptidase RNAs are differentially expressed and show discordant regulation with γ-glutamyl transpeptidase. J. Biol. Chem..

[83] Inamura T., Pardridge W.M., Kumagai Y., Black K.L. (1994). Differential tissue expression of immunoreactive dehydropeptidase I, a peptidyl leukotriene metabolizing enzyme. Prostaglandins Leukot. Essent. Fat. Acids.

[84] Payne R.J.H., Jansen V.A.A. (2001). Understanding bacteriophage therapy as a density-dependent kinetic process. J. Theor. Biol..

[85] Kolonin M.G., Sun J., Do K., Vidal C.I., Ji Y., Baggerly K.A., Pasqualini R., Amp W., Kolonin M.G., Sun J. (2006). Synchronous selection of homing peptides for multiple tissues by in vivo phage display. FASEB J..

[86] Giordano R.J., Lahdenranta J., Zhen L., Chukwueke U., Petrache I., Langley R.R., Fidler I.J., Pasqualini R., Tuder R.M., Arap W. (2008). Targeted induction of lung endothelial cell apoptosis causes emphysema-like changes in the mouse. J. Biol. Chem..

[87] Deng X., Wang L., You X., Dai P., Zeng Y. (2018). Advances in the T7 phage display system (Review). Mol. Med. Rep..

[88] Slootweg E.J., Keller H.J.H.G., Hink M.A., Borst J.W., Bakker J., Schots A. (2006). Fluorescent T7 display phages obtained by translational frameshift. Nucleic Acids Res..

[89] Kaur S., Harjai K., Chhibber S. (2014). Bacteriophage-aided intracellular killing of engulfed methicillin-resistant Staphylococcus aureus (MRSA) by murine macrophages. Appl. Microbiol. Biotechnol..

[90] Zhang L., Sun L., Wei R., Gao Q., He T., Xu C., Liu X., Wang R. (2017). Intracellular Staphylococcus aureus control by virulent bacteriophages within MAC-T bovine mammary epithelial cells. Antimicrob. Agents Chemother..

[91] Lehti T.A., Pajunen M.I., Skog M.S., Finne J. (2017). Internalization of a polysialic acid-binding Escherichia coli bacteriophage into eukaryotic neuroblastoma cells. Nat. Commun..

[92] Dabrowska K., Opolski A., Wietrzyk J., Switala-Jelen K., Boratynski J., Nasulewicz A., Lipinska L., Chybicka A., Kujawa M., Zabel M. (2004). Antitumor activity of bacteriophages in murine experimental cancer models caused possibly by inhibition of β3 integrin signaling pathway. Acta Virol..

[93] Akerman M.E., Chan W.C.W., Laakkonen P., Bhatia S.N., Ruoslahti E. (2002). Nanocrystal targeting in vivo. Proc. Natl. Acad. Sci. USA.

[94] Karmali P.P., Kotamraju V.R., Kastantin M., Black M., Missirlis D., Tirrell M., Ruoslahti E. (2009). Targeting of albumin-embedded paclitaxel nanoparticles to tumors. Nanomed. Nanotechnol. Biol. Med..

[95] Uchida M., Kosuge H., Terashima M., Willits D.A., Liepold L.O., Young M.J., McConnell M.V., Douglas T. (2011). Protein cage nanoparticles bearing the LyP-1 peptide for enhanced imaging of macrophage-rich vascular lesions. ACS Nano.

[96] Soda Y., Marumoto T., Friedmann-Morvinski D., Soda M., Liu F., Michiue H., Pastorino S., Yang M., Hoffman R.M., Kesari S. (2011). Transdifferentiation of glioblastoma cells into vascular endothelial cells. Proc. Natl. Acad. Sci. USA.

[97] Liu K.Y., Yang W.H., Dong X.K., Cong L.M., Li N., Li Y., Wen Z.B., Yin Z., Lan Z.J., Li W.P. (2016). Inhalation Study of Mycobacteriophage D29 Aerosol for Mice by Endotracheal Route and Nose-Only Exposure. J. Aerosol Med. Pulm. Drug Deliv..

[98] Przystal J.M., Waramit S., Pranjol M.Z.I., Yan W., Chu G., Chongchai A., Samarth G., Olaciregui N.G., Tabatabai G., Carcaboso A.M. (2019). Efficacy of systemic temozolomide-activated phage-targeted gene therapy in human glioblastoma. EMBO Mol. Med..

[99] Kropinski A.M., Mazzocco A., Waddell T.E., Lingohr E., Johnson R.P. (2009). Enumeration of bacteriophages by double agar overlay plaque assay. Methods Mol. Biol..

