# Supplementary material for: Modification of Bacteriophages to Increase Their Association with Lung Epithelium Cells In Vitro
Source: Pharmaceuticals (Basel). 2021 Apr 1;14(4):308. doi: 10.3390/ph14040308 (PMC8067280; doi:10.3390/ph14040308)
Supplement: Supplementary file 1 [file pharmaceuticals-14-00308-s001.pdf]

Supplementary Materials:

Table S1. Sequences for the homing peptides used in this study.

Homing peptide	Sequence (aa)	Receptor
GFE-1	CGFECVRQCPERC	membrane dipeptidase [86][68]
GFE-2	CGFELETC	membrane dipeptidase [86] [68]
MTDH domain	GLNGLSSADPSSDWNAPAEEWGNW VDEDRASLLKSQEPISNDQKVSD DDKEKGEGALPTGKSK	lung endothelium putative receptor [80]

Table S2. Sequences used for homologous recombination vector synthesis.

Name	Sequence (bp)
10A (HR arm1)	cgtgctaacttccaagcggaccagattatcgctaagtacgcaatgggccacggtgg tcttcgcccagaagctgctggtgcagtggttttcaaagtggag agcataaccccttggggcctctaaac
10A (HR arm2)	gggtcttgaggggtttttgctgaaaggaggaactatat gcgctcatacgatatgaacgttgagactgccg
10B (HR arm1)	agcataaccccttggggcctctaaacgggtcttgaggggtttttgctgaaaggag gaactatat gcgctcatacgatatgaacgttgagactgccg
10B (HR arm2)	ctagcataaccccttggggcctctaaacgggtcttgaggggtttttgctgaaagga ggaactatatg cgctcatacgatatgaacgttgagactgccg
RBS	aaagaggagaaa
trxA	atgagcgataaaatcattcacctgaccgatgactcttttgataccgacgtgctgaaa gctgatggtgcaattctggttgatttctgggcagagtggtgcggcccttgcaaaatg atcgctccaatcctggacgaaattgcggacgaatatcagggtaagctgactgtggc caaactgaacattgaccagaaccctggcaccgcaccgaaatacggtatccgtggc atcccaactctgctgctgttcaaaaacggtgaagtggcagcaaccaaagtaggcg ctctgtctaaaggccaactgaaagagttcctggacgccaacctggcttaatga
pSMART (IDT)	CCCGTGTAAAACGACGGCCAGTTTATCTAGTCAGCT TGATTCTAGCTGATCGTGGACCGGAAGGTGAGCCAG TGAGTTGATTGCAGTCCAGTTACGCTGGAGTCTGAG GCTCGTCCTGAATGATATGCGGCCGCCGGAGGGTTG CGTTTGAGACGGGCGACAGATATCAGTTCTGGACGA GCGAGCTGTCGTCCGACGCGTGATCTTACGGCATTA TACGTATGATCGGTCCACGATCAGCTAGATTATCTA GTCAGCTTGATGTCATAGCTGTTTCCTGAGGCTCAAT ACTGACCATTTAAATCATACCTGACCTCCATAGCAG

AAAGTCAAAAGCCTCCGACCGGAGGCTTTTGACTTG
ATCGGCACGTAAGAGGTTCCAACTTTCACCATAATG
AAATAAGATCACTACCGGGCGTATTTTTTGAGTTATC
GAGATTTTCAGGAGCTAAGGAAGCTAAAATGAGTAT
TCAACATTTCCGTGTCGCCCTTATTCCCTTTTTTGCGG
CATTTTGCCTTCCTGTTTTTGCTCACCCAGAAACGCT
GGTGAAAGTAAAAGATGCTGAAGATCAGTTGGGTG
CACGAGTGGGTTACATCGAACTGGATCTCAACAGCG
GTAAGATCCTTGAGAGTTTACGCCCCGAAGAACGTT
TTCCAATGATGAGCACTTTTAAAGTTCTGCTATGTGG
CGCGGTATTATCCCGTATTGACGCCGGGCAAGAGCA
ACTCGGTCGCCGCATACACTATTCTCAGAATGACTT
GGTTGAGTACTCACCAGTCACAGAAAAGCATCTCAC
GGATGGCATGACAGTAAGAGAATTATGCAGTGCTGC
CATAACCATGAGTGATAACACTGCGGCCAACTTACT
TCTGGCAACGATCGGAGGACCGAAGGAGCTAACCG
CTTTTTTGCACAACATGGGGGATCATGTAACTCGCCT
TGATCGTTGGGAACCGGAGCTGAATGAAGCCATACC
AAACGACGAGCGTGACACCACGATGCCTGTAGCAA
TGGCAACAACGTTGCGCAAACTATTAACTGGCGAAC
TACTTACTCTAGCTTCCCGGCAACAATTAATAGACT
GGATGGAGGCGGATAAAGTTGCAGGATCACTTCTGC
GCTCGGCCCTCCCGGCTGGCTGGTTTATTGCTGATAA
ATCTGGAGCCGGTGAGCGTGGGTCTCGCGGTATCAT
TGCAGCACTGGGGCCAGATGGTAAGCCCTCCCGCAT
CGTAGTTATCTACACGACGGGGAGTCAGGCAACTAT
GGATGAACGAAATAGACAGATCGCTGAGATAGGTG
CCTCACTGATTAAGCATTGGTAATGAGGGCCCAAAT
GTAATCACCTGGCTCACCTTCGGGTGGGCCTTTCTTG
AGGACCTAAATGTAATCACCTGGCTCACCTTCGGGT
GGGCCTTTCTGCGTTGCTGGCGTTTTTCCATAGGCTC
CGCCCCCCTGACGAGCATCACAAAAATCGATGCTCA
AGTCAGAGGTGGCGAAACCCGACAGGACTATAAAG
ATACCAGGCGTTTCCCCCTGGAAGCTCCCTCGTGCG
CTCTCCTGTTCCGACCCTGCCGCTTACCGGATACCTG
TCCGCCTTTCTCCCTTCGGGAAGCGTGGCGCTTTCTC
ATAGCTCACGCTGTAGGTATCTCAGTTCGGTGTAGGT
CGTTCGCTCCAAGCTGGGCTGTGTGCACGAACCCCC
CGTTCAGCCCGACCGCTGCGCCTTATCCGGTAACTA
TCGTCTTGAGTCCAACCCGGTAAGACACGACTTATC
GCCACTGGCAGCAGCCACTGGTAACAGGATTAGCA
GAGCGAGGTATGTAGGCGGTGCTACAGAGTTCTTGA
AGTGGTGGCCTAACTACGGCTACACTAGAAGAACA
GTATTTGGTATCTGCGCTCTGCTGAAGCCAGTTACCT
CGGAAAAAGAGTTGGTAGCTCTTGATCCGGCAAAC
AAACCACCGCTGGTAGCGGTGGTTTTTTTGTTTGCAA

	GCAGCAGATTACGCGCAGAAAAAAAGGATCTCAAG AAGATCCTTTGATTTTCTACCGAAGAAAGGCCCA

Table S3. Primers used in this study.

Primer	Sequence (bp) 5′ to 3′
HP-fwd-1	agaacaaggccgcacttaccga
HP-fwd-2	aaggagattattgcggctctgactaa
HP-rev-1	tcagcggcagtctcaacgtt
HP-rev-2	tagacgccagaatgtcgttcaca
